# Altering Terahertz Sound Propagation in a Liquid upon Nanoparticle Immersion

**DOI:** 10.3390/nano12142401

**Published:** 2022-07-14

**Authors:** Alessio De Francesco, Ferdinando Formisano, Luisa Scaccia, Eleonora Guarini, Ubaldo Bafile, Marco Maccarini, Dmytro Nykypanchuck, Alexei Suvorov, Yong Q. Cai, Scott T. Lynch, Alessandro Cunsolo

**Affiliations:** 1 CNR-IOM & INSIDE@ILL c/o Operative Group in Grenoble (OGG), F-38042 Grenoble, France; formisan@ill.fr; 2 Institut Laue-Langevin (ILL), F-38042 Grenoble, France; 3 Dipartimento di Economia e Diritto, Università di Macerata, Via Crescimbeni 20, I-62100 Macerata, Italy; scaccia@unimc.it; 4 Dipartimento di Fisica e Astronomia, Università di Firenze, Via G. Sansone 1, I-50019 Sesto Fiorentino, Italy; guarini@fi.infn.it; 5 Consiglio Nazionale delle Ricerche, Istituto di Fisica Applicata “Nello Carrara”, Via Madonna del Piano 10, I-50019 Sesto Fiorentino, Italy; u.bafile@ifac.cnr.it; 6 Université Grenoble-Alpes, CNRS, UMR 5525, VetAgro Sup, Grenoble INP, TIMC, F-38000 Grenoble, France; marco.maccarini@univ-grenoble-alpes.fr; 7 Energy & Photon Sciences Directorate, Brookhaven National Lab, Upton, NY 11973, USA; dnykypan@bnl.gov (D.N.); asuvorov@bnl.gov (A.S.); cai@bnl.gov (Y.Q.C.); 8 Department of Physics, University of Wisconsin at Madison, 1150 University Avenue, Madison, WI 53706-1390, USA; slynch6@wisc.edu

**Keywords:** Inelastic X-ray Scattering, phonon propagation, nanoparticles, model choice, Bayesian inference

## Abstract

One of the grand challenges of new generation Condensed Matter physicists is the development of novel devices enabling the control of sound propagation at terahertz frequency. Indeed, phonon excitations in this frequency window are the leading conveyor of heat transfer in insulators. Their manipulation is thus critical to implementing heat management based on the structural design. To explore the possibility of controlling the damping of sound waves, we used high spectral contrast Inelastic X-ray Scattering (IXS) to comparatively study terahertz acoustic damping in a dilute suspension of 50 nm nanospheres in glycerol and on pure glycerol. Bayesian inference-based modeling of measured spectra indicates that, at sufficiently large distances, the spectral contribution of collective modes in the glycerol suspension becomes barely detectable due to the enhanced damping, the weakening, and the slight softening of the dominant acoustic mode.

## 1. Introduction

The control of sound propagation through the design of the mesoscale structure is one of the new frontiers in Condensed Matter Physics. This goal becomes especially compelling at terahertz frequencies, where phonons become the leading conveyors of heat transfer in insulators, their manipulation being critical for developing a whole new class of devices implementing heat flow management based upon nanoscale design [1]. Along this route, a crucial concern is the control of the damping of acoustic waves which, ultimately, is likely to have a visible impact on heat flow regulation.

From a complementary perspective, the origin and driving mechanism of acoustic damping in disordered materials is also a topic of fundamental interest that has challenged Condensed Matter physicists for decades [2,3,4]. In general, how a liquid resists sound propagation depends on the dynamic window; at low frequencies, viscous dissipative processes such as diffusion and relaxation play a primary role in sound damping [5]. Conversely, over the short times and distances characteristic of the elastic regime of a viscoelastic fluid [6], both viscous flow and structural relaxations are frozen, and the dynamic response of a liquid resembles that of its solid counterpart [7]. Generally speaking, in amorphous systems, the damping of density fluctuations primarily arises from two contributions:

(1) Elastic constants anisotropy

The damping effect of elastic constants anisotropy can be easily understood by representing an amorphous system as an ensemble of micro-crystallites, small enough to elude detection by conventional diffraction yet large enough to include a few shells of neighboring atoms. The mere existence of a local ordering disrupts the elastic isotropy of the medium, making specific directions more favorable to sound propagation than others. Hence, a density fluctuation propagating through multiple randomly-oriented crystallites acquires a variety of sound velocities, thus getting fragmented into different acoustic modes whose mutual interference ultimately enhances the sound damping.

(2) Structural disorder

The intrinsic disorder of an amorphous material can be conceptualized as the superposition of “inherent” structures characterized by distinct sets of local lattice vectors defining the internal lattice of micro-crystallites. These vectors change both in direction—reflecting the random micro-crystallites orientation—and in magnitude—due to the lack of well-defined spatial periods (interatomic distances). Because sound propagation is interconnected with atomic separations, fluctuations of lattice distances finally cause variations in sound speed. Density fluctuations propagating through such a system are redistributed again into multiple acoustic modes having different speeds, whose mutual interference favors acoustic damping.

In summary, the high-frequency sound damping in a liquid reflects the lack of global symmetry (anisotropy) and periodicity (spatial repetition) of lattice sites.

Thus, far we have assumed that probed timescales are so short that viscous rearrangements appear frozen and the damping mechanism in liquids resembles its counterpart in glasses. Over longer timescales, acoustic damping becomes more complex in a liquid, as diffusion and relaxation processes become increasingly relevant.

Even more complex is the case of colloidal suspensions; in these systems, the damping mechanism [8,9,10,11,12,13] is dominated by elastic heterogeneity, i.e., by the elastic constants mismatch between floating colloids and hosting medium. Like in the cases mentioned above, this inhomogeneity causes sound speed fluctuations, which ultimately reduce the acoustic mode lifetime. A more subtle effect of this heterogeneity stems from the different rigidity of the NPs and the surrounding fluid, due to their different expansion/contraction when crossed by a compression-rarefaction (acoustic) wave. Owing to this unmatched expansion, shear stresses develop at the colloids interface and they enhance the system resistance to sound propagation. Finally, another damping contribution in colloidal systems arises from the scattering of acoustic waves at the interface between the nanoparticles and the hosting liquid. Again these reflections change the phase of sound waves.

Our previous measurements [11,12] suggested that one can significantly enhance sound damping via the immersion of even sparse amounts of nano-objects. Indeed, this strategy visibly changes the high-frequency acoustic damping probed, e.g., by Inelastic X-ray (IXS) methods [14,15].

In a typical IXS measurement, a beam of X-ray photons with energy far from any sample resonance impinges on a sample at rest and in thermal equilibrium. The spectral density of photons scattered by the sample is then scanned within a few THz, i.e., over frequencies smaller than that of incident photons by seven orders of magnitude [14]. Towards the dawn of the new millennium, the enhanced brilliance of third-generation synchrotron sources and parallel advances in crystal optics fabrication endowed IXS spectrometers with the resolving powers and count rates needed to perform this task. This improved performance paved the way for novel investigations of acoustic excitations in noncrystalline materials with wavelengths and frequencies approaching first neighboring atom separations and “cage oscillation” frequencies, respectively. Nowadays, IXS represents an obvious choice to test the feasibility of terahertz sound manipulation in nanostructured materials.

Owing to the still pioneering nature of these investigations, it is desirable to deal with simple prototypical systems; sensible candidates are nanospheres immersed in a liquid in such a sparse amount to be reasonably assumed as non-interacting. Furthermore, gold seems especially fit as nanoparticle material because its monatomic and crystalline nature makes the interpretation of its spectral contribution more straightforward. Finally, a spherical shape is advisable to eliminate its possible rotational contribution to the scattered intensity.

In an IXS measurement on such colloidal suspension, a high spectral contrast is needed to reliably characterize the generalized acoustic modes, whose spectral signature is frequently partially obscured by the wings of the (resolution-convoluted) dominant elastic peak. In this respect, the 10ID beamline [16] of the National Synchrotron Light Source II represents an invaluable resource for IXS studies of this kind, owing to its worldwide unrivaled spectral contrast.

In this work, we decided to use this spectroscopic tool to measure the IXS scattering from a diluted (less than 1% in volume) suspension of spherical 50 nm diameter gold nanoparticles (Au-NPs) in glycerol. As a reference, we compare IXS spectra from the suspension with those of pure solvent (glycerol), both samples being kept at ambient conditions. Spectral shapes are analyzed using a Bayes inferential method already described, e.g., in Refs. [17,18]. This method enables a minimally biased and evidence-based probabilistic assessment of the most plausible number of inelastic modes contributing to the spectrum, and of the distribution probabilities of model parameters [11,13,19,20].

## 2. Materials and Methods

Inelastic X-ray Scattering measurements were executed at the high-resolution beamline 10ID of the National Synchrotron Light Source-II at Brookhaven National Laboratory [16]. The instrumental resolution profile is about 2 meV broad with sharp, nearly Gaussian tails. Moreover, its superior resolution in the wavevector transfer *Q* provides access to *Q*’s as low as 0.5 nm^−1^. The incident beam energy is around 9.13 keV, with the energy analysis being performed by rocking the crystals of the monochromator unit while keeping the analyzer optics fixed. Such a scan was alternatively implemented by the simultaneous rocking of two crystal pairs arranged in the so-called four-bounce design. The analyzer unit is mounted at the extreme of a spectrometer arm. It is based on a collimator-(energy) disperser-wavelength (selector), or CDW, optical design and coupled with an upstream collimating mirror which enhances its angular acceptance. The 10ID spectrometer is described in further detail in Ref. [16]. IXS spectra were collected in the 1.3 nm^−1^ ≤ *Q* ≤ 4.6 nm^−1^ intervals and approximately covered the −15 meV ÷ +15 meV energy window.

## 3. Results

**Figure 1:**
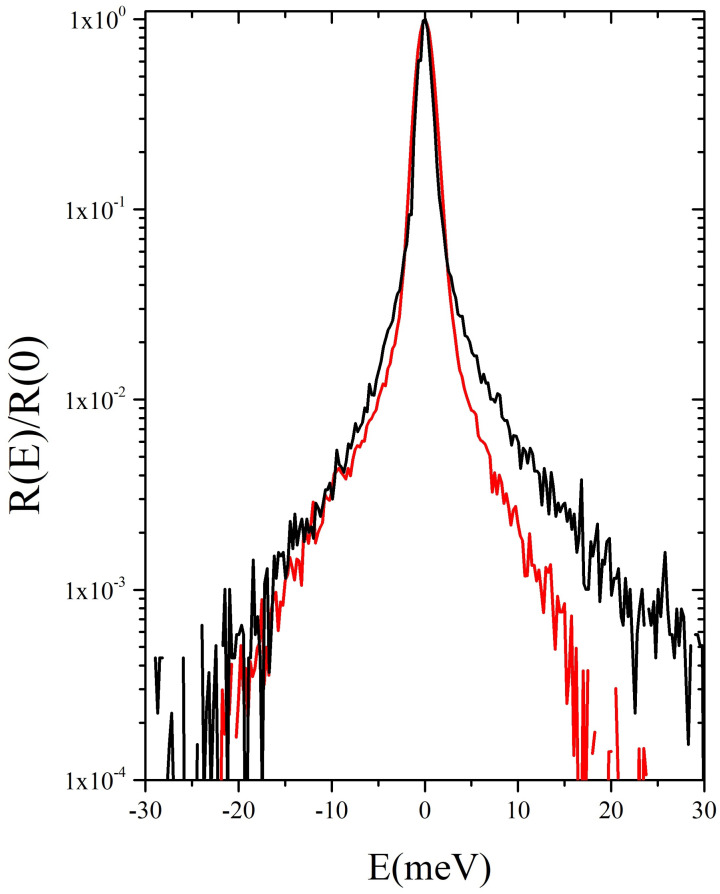
The instrumental energy resolution of the current measurement (red line) is here compared with that of a typical state of the art IXS spectrometer, Sector 30 at the Advanced Photon Source in Argonne National Laboratory (black line). For the sake of comparison, both resolution lineshapes R(E) are normalized to the respective maxima R(0).

As discussed above, one of the primary motivations of the present work is to investigate with a superior spectral contrast the effect of immersed nanoparticles on the collective modes of a colloidal suspension. Indeed, in a previous measurement [12], we measured the IXS spectra of diluted suspensions of Au-NPs (50 and 200 nm in diameter) in glycerol exploiting one of the state of the art IXS spectrometers: the Sector 30 beamline [21,22] at the Advanced Photon Source (Argonne National Laboratory, Chicago, IL, USA). Figure 1 provides a demonstration of the spectrometers performance by comparing the energy resolution profile of the current measurement with the one of the Sector 30 beamline [23,24]. For the sake of comparison, both lineshapes are normalized to the respective maxima. One can readily notice that, although the latter lineshape is slightly narrower, its wings have a substantially slower frequency-decay, at least on the Stokes side. The enhanced sharpness of the 10ID resolution ensures that the inelastic features of interest for the current measurement, whose position ranges in the 2 meV ≤ ℏω ≤ 10 meV window, are unlikely to be dramatically overshadowed by the tails of the dominating central peak.

**Figure 2:**
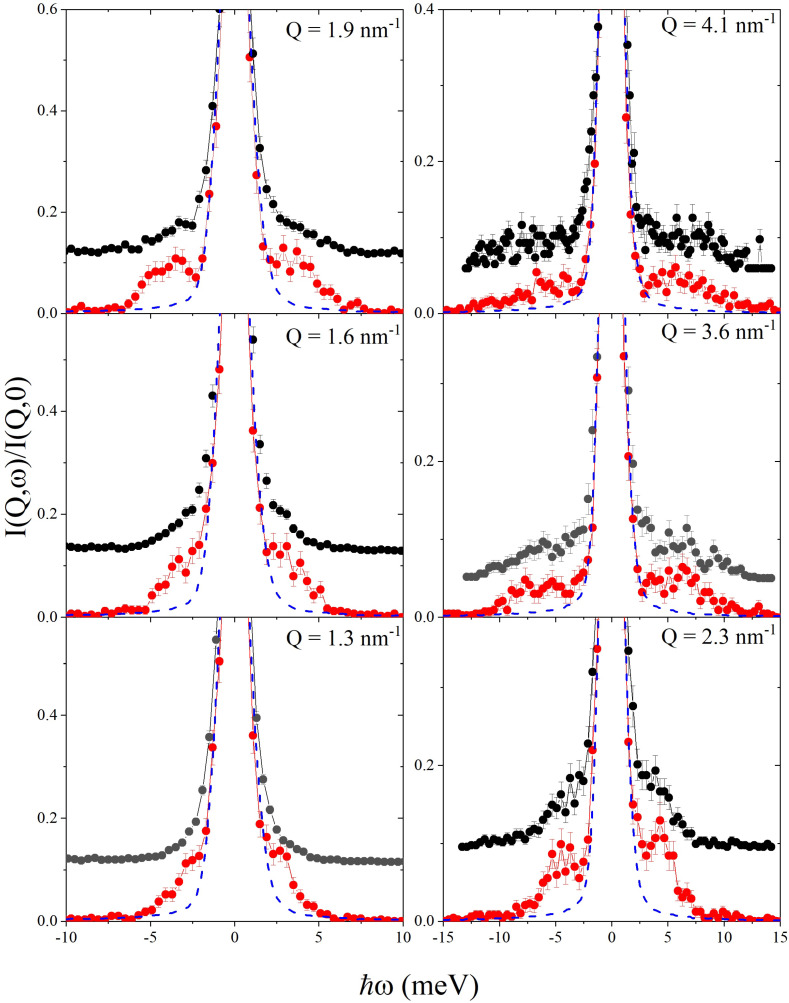
Inelastic X-ray Scattering (IXS) spectra measured at the indicated wave wavevector transfer *Q* values on pure glycerol (red dots) and on the Au-NP suspension in glycerol (black dots) are reported along with the instrumental energy resolution function (dashed blue lines). All lineshapes are normalized to the respective maximum intensity; those from the nanoparticle suspension are also vertically shifted for clarity.

In Figure 2, IXS spectra measured at representative *Q*’s either on the Au-NP glycerol suspension or in pure glycerol are compared with each other and with the energy resolution profiles. All lineshapes are normalized to their maximum intensity, with the suspension ones being also shifted vertically for clarity. All scattering profiles display the expected triplet structure comprising a dominant central peak and two side shoulders, slightly asymmetrical because of the well-known detailed balance principle. These, respectively, arise from non-propagating density fluctuations, like microscopic diffusions and structural relaxations, or propagating ones, i.e., acoustic-like modes. Indeed, the latter excitations have an inelastic shift that systematically grows upon *Q*-increase. However, immersed Au-NPs leave an evident signature on the inelastic shoulders, which appear significantly less pronounced than their counterparts in pure glycerol. This trend is more noticeable at low *Q*’s, especially at the lowest one, where inelastic features in the Au-NP suspension spectra, if present at all, are entirely hidden by the resolution wings.

At this stage, even basic phenomenological aspects of the observed overall trend still appear mostly elusive. For instance, it is still unclear if the less structured inelastic wings of the suspension spectra are secondary to the enhancement of either the damping, the attenuation, or the softening of the acoustic mode or, possibly, to a combination of these effects, which, respectively, cause a broadening, a weakening, and a red-shift of the dominant inelastic mode. To cast these qualitative observations on a more quantitative basis, we decided to perform a Bayesian inference-based analysis of the measured spectra, as described in previous works in some detail [17,18,20]. The measured spectral shapes were approximated with a model including an *a priori* unknown finite number of Damped Harmonic Oscillator (DHO) profiles [25], complemented by a Dirac δ-function δ(E) to account for the elastic portion of the measured signal. The choice of the most appropriate model profile to approximate the spectral shape of a hybrid, solid–liquid system is far from trivial since no current theory can provide a firm reference. However, the dilute nature of the nanoparticle concentration suggests that the scattering from glycerol provides the dominant portion of the spectral shape. As customary for glass-forming materials [4], the central peak of the spectrum was assumed to be the infinitely narrow fingerprint of slow internal rearrangements, thus resembling a Dirac δ-function. Furthermore, simple DHO pairs have been identified as the most realistic approximations of the excitation profiles in solid-like media as glycerol and, even more, crystalline gold [4,26,27]. In summary, the spectrum of density fluctuation, i.e., the dynamic structure factor S(Q,E) was expressed as:

(1) $$\begin{array}{c} S\left(Q,E\right)=A_{e}\delta \left(E\right)+\left[n\left(E\right)+1\right]\frac{E}{k_{B}T}\\ \times \left\{\sum_{i=1}^{k}\frac{2}{\pi }A_{i}{\mathrm{DHO}}_{i}\left(Q,E\right)\right\},\; \end{array}$$

where $A_{e}$ represents the area of the elastic peak, E=ℏω is the energy exchanged between the probe particles and the system and each of the ${\mathrm{DHO}}_{i}$ terms accounts for an inelastic excitation in the spectrum. The energy-dependent term $n\left(E\right)={\left(e^{\hbar \omega /k_{B}T}-1\right)}^{-1}$ represents the Bose factor which expresses the detailed balance condition, with $k_{B}$ being the Boltzmann constant and *T* the sample temperature. The *i*-th DHO*_i_* profile in Equation (1) has undamped frequency $\Omega_{i}$, damping coefficient $\Gamma_{i}$ and is multiplied for an intensity factor $A_{i}$. All spectral parameters in Equation (1) are in principle *Q* dependent, although, in the used notation, we dropped the explicit mention of such a dependence. Noticeably, the number, *k*, of DHO profiles included in the model is here a free parameter whose optimal value is to be determined by the Bayesian inference algorithm conditional on the measurement outcome.

A standard model of the measured lineshape should also account for the various experimental factors affecting the spectral measurement, including the environmental background and the instrumental resolution line-broadening. In practice, the model ultimately fitting each scattering profile has the following form:

(2) I(Q,E)=R(E)⊗S(Q,E)+B(E)

where ⊗ represents the convolution operator, R(E) the instrumental energy resolution function, while B(E) is a linear term accounting both for the spectral background and the electronic noise of the detectors. The conducted inferential analysis, indicated the higher plausibility of the one-DHO (i.e., *i* = 1) option. According to the adopted notation, this inelastic profile, its undamped frequency, and damping coefficient will be labeled by the index “1”, and it will be the main focus of the following discussion.

**Figure 3:**
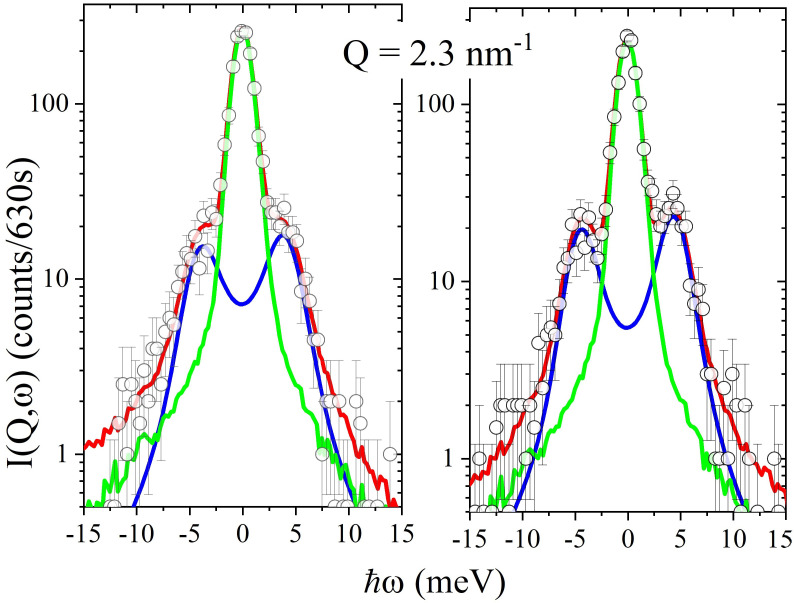
Two IXS spectra measured either in the Au–NP suspension and pure glycerol are reported in the left and right plots, respectively, and therein compared with best fitting model lineshapes (in red) obtained as discussed in the text. The resolution convoluted Damped Harmonic oscillator (blue line) and the elastic contribution δ(E) (green line) model components are also reported for reference.

Figure 3 compares a spectrum measured in either the suspension or in the pure solvent to the corresponding most plausible model lineshape along with its elastic and inelastic components. The plot demonstrates that this simple model option, which contains a single DHO profile, DHO_1_, accurately describes the scattering profile from both samples. After assessing this model performance, we can focus on the *Q*-dependence of optimal values of its relevant parameters, namely $\Omega_{1}$, $\Gamma_{1}$, and $A_{1}$.

## 4. Discussion

**Figure 4:**
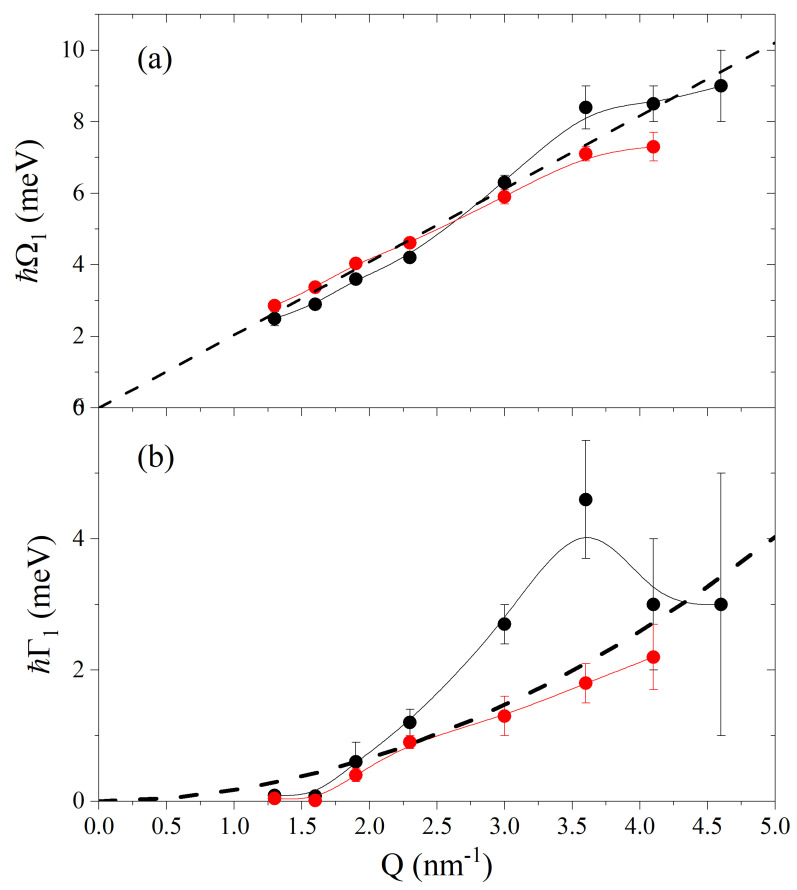
Panel (**a**): the *Q*–dependence of the undamped frequency, $\Omega_{1}$, of the DHO model profile (see text) is reported as obtained from the best–fitting of IXS spectra of the Au–NP suspension in glycerol (black dots) and pure glycerol (red dots). The solid line through experimental values are splines serving as guides to the eye. The linear dispersion expected in the elastic regime $c_{\inf}Q$ is also reported (dashed black line) for reference, as derived using the elastic, or infinite frequency, sound velocity $c_{\inf}$ reported in Ref. [27]. Panel (**b**): the corresponding best–fit values of the DHO half–width $\Gamma_{1}$ are reported with the same symbol together with the quadratic *Q* dependence (black dashed line) as derived from Ref. [4].

The *Q*-dependence of the inelastic shift $\Omega_{1}$ extracted from the best-fit of suspension and pure glycerol spectra are reported in Figure 4a and therein compared with the linear dispersion $c_{\inf}\cdot Q$ expected in the elastic, or high-frequency, limit. Here, $c_{\inf}$ represents the infinite frequency sound velocity, i.e., the sound propagation speed attained by a viscoelastic fluid in the elastic limit, as glycerol is at *Q* values probed in this measurement. Its value was derived from Ref. [27]. The overall *Q*-trend of the $\Omega_{1}$ values is consistent with that typically observed in glycerol, with the linear dispersive regime being approached in both samples from low to moderate *Q*’s [4]. However, at low *Q* values, $\Omega_{1}$ values seem slightly, yet systematically, lower for the suspension sample. The values of the damping coefficient $\Gamma_{1}$ shown in Figure 4b clearly demonstrate that these are systematically higher in the Au-NP suspension than in pure glycerol for Q> 2 nm^−1^. The systematically larger values of the damping coefficient $\Gamma_{1}$ in the suspension data confirm our previous findings and demonstrate that even sparse amounts of NPs in immersion significantly reduce the lifetime of collective modes propagating in a liquid [11,12].

**Figure 5:**
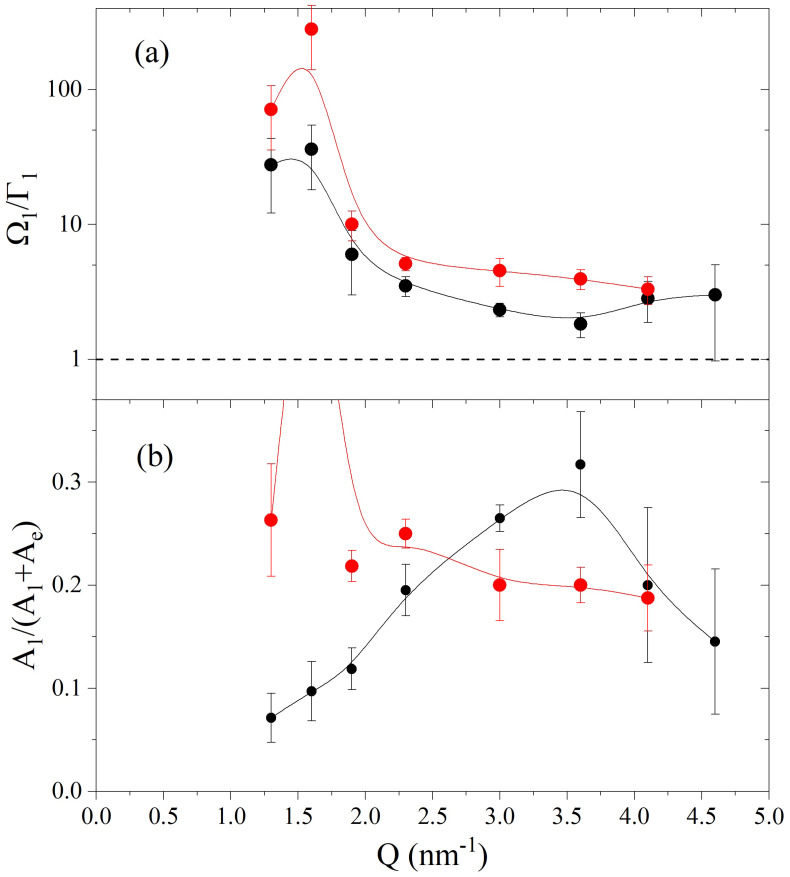
Panel (**a**) displays the *Q*–dependence of the inverse of the relative damping $\Omega_{1}/\Gamma_{1}$ of the best–fitting DHO model profile (see text). Panel (**b**) shows the area of the DHO component relative to the one of the entire best fitting model. Symbols and colors are as in Figure 4. Notice that the *Q* = 1.5 nm^−1^ value falls well beyond the plot window.

Further insight into this effect is provided in Figure 5 which reports the inverse of the relative damping $\Omega_{1}/\Gamma_{1}$ (panel a) and the relative amplitude of the inelastic mode $A_{1}/\left(A_{1}+A_{e}\right)$ (panel b). It readily appears that the excitation in the suspension is significantly closer to the critical damping, $\Omega_{1}/\Gamma_{1}=1$, than its counterpart in pure glycerol, even though this difference vanishes for the highest *Q*’s reached by this measurement. It appears that for *Q* values lower than about 2.5 nm^−1^ the relative amplitude of the inelastic mode in the suspension is systematically lower than for pure glycerol, although the opposite seems to be true for 2.5 nm^−1^ < *Q* < 4 nm^−1^.

**Figure 6:**
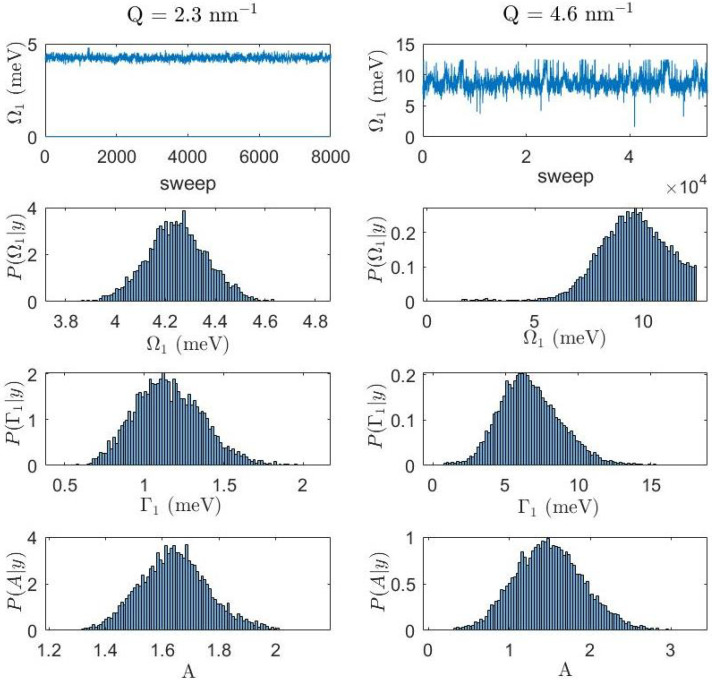
The trace plot of $\Omega_{1}$ (upper two panels) and the posterior distribution of the various ${\mathrm{DHO}}_{1}$ parameters for the Au–NP suspension, are reported for the two indicated *Q* values; the posterior distributions reported in the ordinate axis are nothing but the conditional probability of a given model parameter having observed the collected experimental data *y*.

Besides the improved spectrometer performance, a critical asset of this study is the opportunity of performing minimally biased analyses of measured spectra. In this approach, the most plausible number of inelastic excitations contributing to the scattering profile is determined probabilistically conditional to the experimental outcome and in compliance with the so-called Occam razor principle, or “*lex parsimoniae*”. The latter principle states that, among equally plausible competing models, the one containing the smallest number of free parameters is always to be preferred [28]. Most importantly, the used approach identifies, conditional to the collected experimental data, not only the optimal parameter values but also the probability distribution of each parameter. To this scope, a reversible jump Monte Carlo algorithm [29] is used to sweep the hyperspace of all free parameters, each parameter posterior being ultimately drawn by counting the times the various parameter values were visited by the algorithm. The outcome of the present study unambiguously indicates that a single excitation is the most plausible hypothesis on the spectrum of glycerol in the explored range, although the alternative double excitation hypothesis acquires non-negligible probability at the two highest *Q*’s. In this respect, current results are not incompatible with our previous study on glycerol in a larger *Q* interval suggesting the onset of an additional low-frequency contribution ascribed to interface waves [12]. Figure 6 displays, for two exemplary *Q* values, the posterior distributions drawn for the ${\mathrm{DHO}}_{1}$ parameters and the trace plots of $\Omega_{1}$, i.e., the $\Omega_{1}$ values visited as a function of the algorithm sweeps. One can readily appreciate the unimodal and fairly symmetric shape of various posterior distributions, as well as the typical “hairy caterpillar” trend the trace plots have when the Markov Chain Monte Carlo (MCMC) algorithm, described in depth in Ref. [17], is testing correctly the parameter space. All these trends combined strongly endorse the plausibility of the single excitation model option recommended by the algorithm.

## 5. Conclusions

In conclusion, the presented work is part of a long-term effort towards the control of terahertz phonon propagation through the design of the nanoscale structure. This topic is at the forefront of Condensed Matter Physics and is deemed to profoundly impact heat flow management, as heat transport in insulators mainly uses terahertz phonons as conveyors. As preliminary steps in this emerging field, we focused on the damping effect of immersed nanoparticles on the acoustic modes of the hosting liquid. In this endeavor, we took advantage of the outstanding spectral contrast of a new Inelastic X-ray Scattering beam recently developed at Brookhaven National Laboratory to jointly measure the spectrum of density fluctuations in a diluted suspension of gold nanospheres in glycerol and in the pure solvent. The use of a Bayesian inference based analysis of measured spectral shapes confirms that nanoparticles in immersion have a visible impact on the propagation of collective modes in the hosting liquid. More specifically they systematically enhance the damping of terahertz acoustic modes at least in a well defined wavevector *Q* region, where this mode is clearly discernible in the spectrum. Furthermore, from low to moderate *Q*’s immersed nanoparticles significantly suppress the spectral contribution of this mode, eventually leading to its complete disappearance for distances $\leq 2\pi /Q^{-1}\approx$ 6 nm. We ascribe this effect to the large scattering that propagating density waves experience at the colloid interface, which causes a loss of coherence in the sound propagation. Although significant challenges keep us apart from the goal of sound engineering, we believe that the presented results are both meaningful and encouraging. Indeed, most popular attempts to implement sound manipulation are complementary to the one proposed here, as they rely on the development of phononic crystals [30]. These are artificial nanostructures whose periodicity interferes destructively with the propagation of sound waves of specific wavelengths, slowing, reflecting, or even trapping them inside propagation gaps. Engineering these materials requires special care in maintaining a global periodicity of the mesoscale structure. In particular, the development of devices active in the nearly uncharted terahertz range primarily rests on perfectly ordered quasi-macroscopic superlattices having nanometer spacing, while this objective still poses significant technical challenges, our results suggest that terahertz sound propagation can be effectively shaped using disordered heterogeneous media. Wherever this route will bring us in the long-term effort of sound manipulation, we believe it is worth thorough consideration. A further experimental effort would help to clarify this effect dependence on colloids characteristics, including shape, size and concentration, as well as their mutual interactions.
